# Supplementary material for: C1q/TNF-Related Protein 3 Prevents Diabetic Retinopathy via AMPK-Dependent Stabilization of Blood–Retinal Barrier Tight Junctions
Source: Cells. 2022 Feb 23;11(5):779. doi: 10.3390/cells11050779 (PMC8909652; doi:10.3390/cells11050779)
Supplement: Supplementary file 1 [file cells-11-00779-s001.zip › cells-1593130-supplementary.pdf]

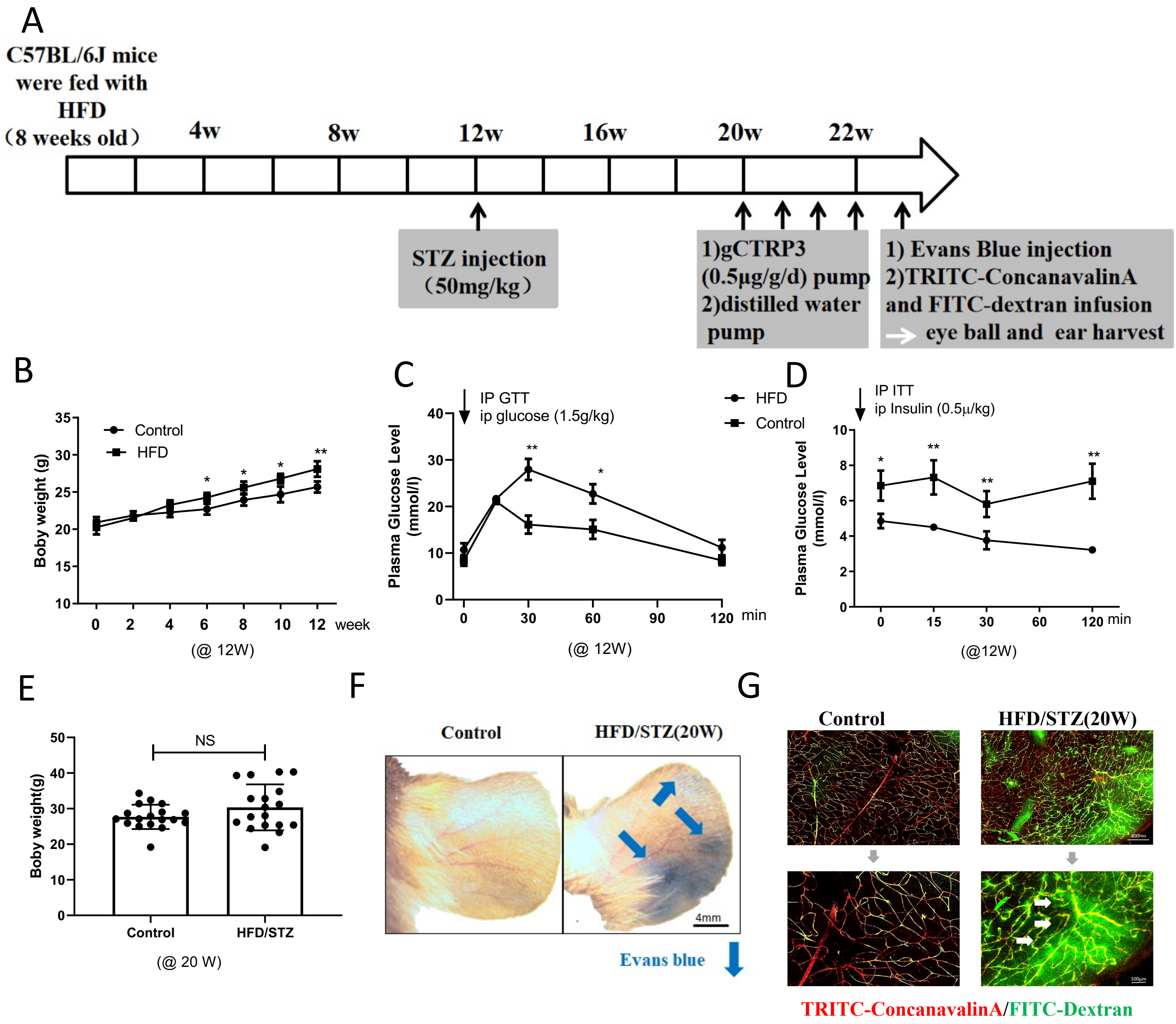

Supplemental Figure S1. The characterization of Diabetic retinopathy mice model
A: Schematic illustration of the experiment flow. B: The body weights of diabetic C57BL/6J mice. C, D: The glucose tolerance test and insulin tolerance test revealed that diabetic mice exhibiting abnormal glucose tolerance and insulin resistance. E: No significant difference in body weight between the vehicle-treated and T2D group on 20 weeks. n = 18. F: Leakage of Evans blue dye to the ear adjacent tissue on diabetic retinopathy mice. G: Confocal images of the retinal tissue showing the retinal vascular leakage. Up, 200x magnificence images; Down, 400x magnificence images. Data are means ± SD. *P < 0.05, **P < 0.01, ***P < 0.001; NS, not significant. gCTRP3, globular domain isoform of C1q/tumor necrosis factor–related protein-3; HFD: high-fat diet; STZ: streptozotocin; Blue arrows indicate ear capillary leakage. White arrows indicate retinal vascular leakage.

Supplemental Figure S1

A

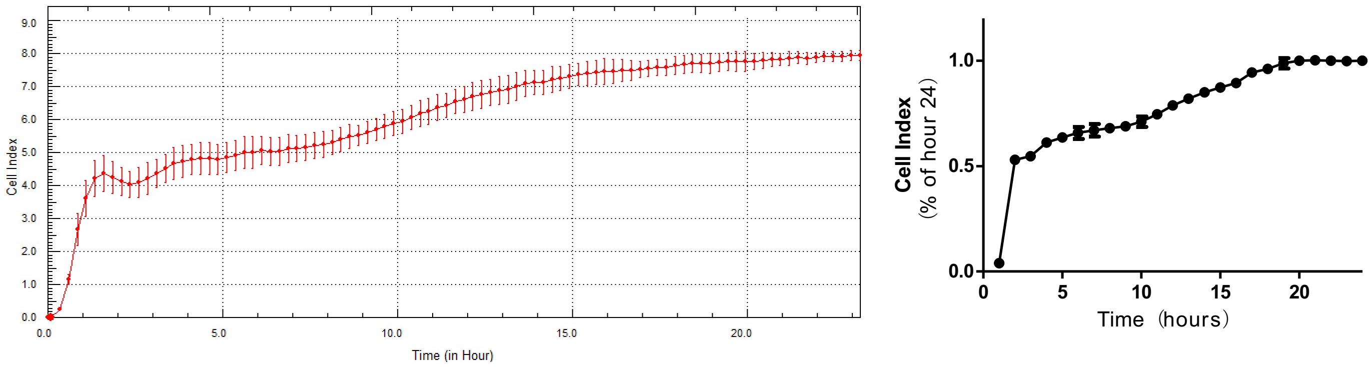

B

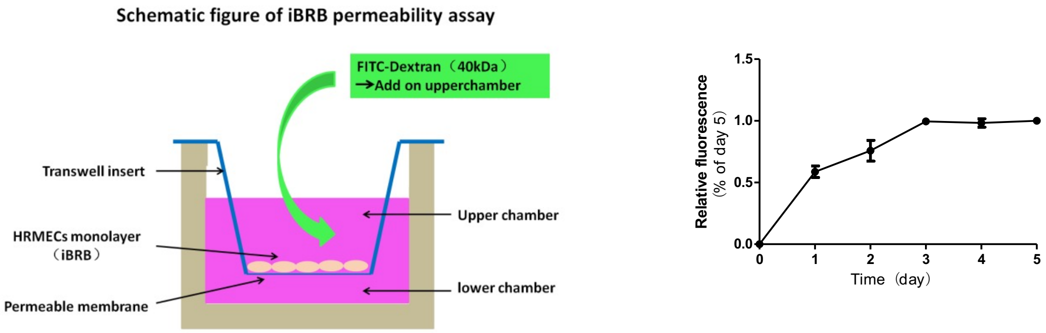

Supplemental Figure S2. The baseline of techniques of xCELLigence and Schematic Figure of Transwell permeability assay. A: The Baseline and quantification of Cell index for xCELLigence assay. B: Schematic illustration of the experiment of iBRB permeability assay.Data are means ± SD.n=6-8, *P < 0.05, **P < 0.01, NS, not significant.

Supplemental Figure S2

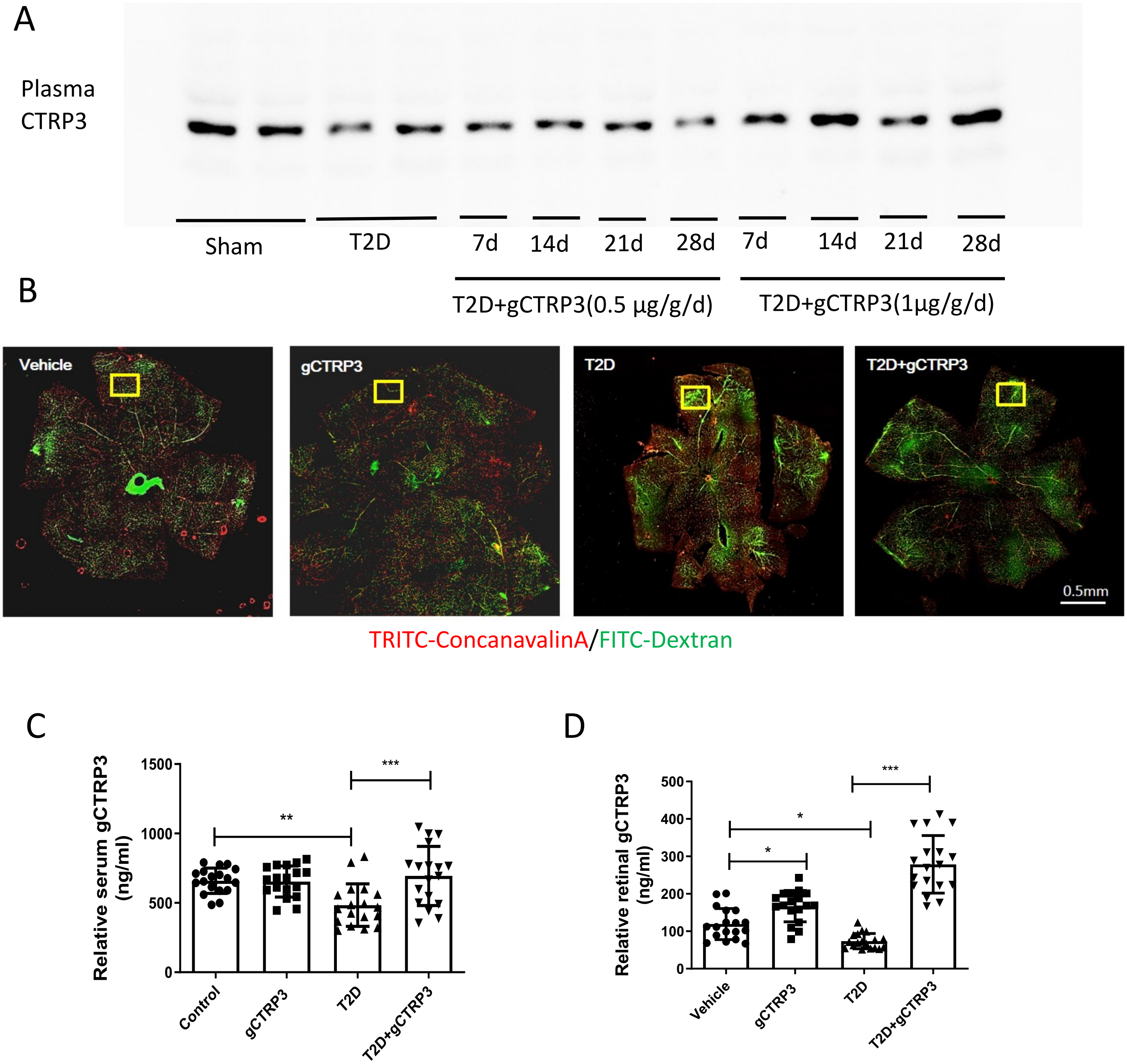

Supplemental Figure S3. The characterization of mice model. A: Effects of CTRP3 supplement upon plasma CTRP3 level evaluated by Western analysis. (n=5). B:All micrographs are merged confocal images of the whole retinal flat mounts. The yellow box is the intercepted part of Figure2A. C: plasma concentration of CTRP3 was measured by ELISA. D: The concentration of CTRP3 was evaluated by ELISA in retinal tissue. n = 18. Data are means ± SD. *$P < 0.05$, **$P < 0.01$, ***$P < 0.001$; NS, not significant.

Supplemental Figure S3
